# Cumulative Exposure to Neighborhood Conditions and Substance Use Initiation among Low-Income Latinx and African American Adolescents

**DOI:** 10.3390/ijerph182010831

**Published:** 2021-10-15

**Authors:** Eunice Lee, Anna Maria Santiago

**Affiliations:** 1 Jack, Joseph and Morton Mandel School of Applied Social Sciences, Case Western Reserve University, 10900 Euclid Avenue, Cleveland, OH 44106, USA; 2 College of Social Science, Michigan State University, 509 East Circle Drive, 224 Berkey Hall, East Lansing, MI 48824, USA; santia63@msu.edu

**Keywords:** neighborhood social disorder, cumulative risk and resilience, adolescence, substance use initiation

## Abstract

Purpose: While previous research underscores the important role that neighborhood contexts play for child and adolescent health and well-being, how these neighborhood contexts influence substance use initiation among adolescents from low-income and ethnic minority families has been understudied. Methods: This study is a secondary analysis of data from the *Denver Child Study* a retrospective survey that uses a natural experiment aimed at assessing neighborhood effects on developmental outcomes of Latinx and African American adolescents (N = 736). Cox cause-specific hazards models were estimated to test: (1) the effects of cumulative exposure to neighborhood social disorder, neighborhood violent and property crime rates, and neighborhood social capital during preadolescence (ages 8–11) on the likelihood of initiating alcohol, cigarette, and marijuana use during adolescence (ages 12–18), after controlling for youth, caregiver, and household factors; and (2) whether the effects of these cumulative neighborhood factors vary by Latinx and African American ethnicity. Results: 5.6% of adolescents in this study initiated cigarette use, 5.4% initiated alcohol use and 5.2% used marijuana for the first time during adolescence. The results indicate that exposure to neighborhood social disorder during preadolescence is a significant risk factor, especially for the initiation of cigarette use (HR = 1.36, 95% CI = 1.062–1.745, *p* = 0.015) particularly among Latinx adolescents (HR = 1.42, 95% CI = 1.031–1.966, *p* = 0.032). Conclusions: The findings suggest the need for further research on the relationship between exposure to neighborhood social disorder and adolescent substance use initiation in order to develop and implement community-based prevention and intervention programs to reduce substance use initiation and facilitate healthy adolescent development.

## 1. Introduction

Adolescent substance use in the United States is associated with a wide variety of deleterious developmental outcomes. Studies have linked substance use during adolescence to delinquency [1], risky sexual behavior [2], and sexual victimization [3]. Substance using adolescents exhibit poorer academic performance at school [4,5] and higher rates of school drop-out [6] than their non-using peers. Adolescent substance use also has been associated with adverse health outcomes, including substance abuse and dependence in later adulthood and some forms of cancer which, in turn, can contribute to premature mortality [7,8,9].

Adolescence is a critical developmental period for initiating health-related risk behaviors such as alcohol, cigarette, and marijuana use. In 2019, substance use initiation rates for U.S. adolescents aged 12 to 17 years were 9.1% for alcohol, 2.3% for cigarettes, and 5.4% for marijuana [10]. Although initiation rates for these substances have decreased in recent years, these substances have become more easily accessible to youth [11]. Additionally, a sizable proportion of American adolescents perceive no great risk from using these substances. More than one-third of youth perceive no great risk from having four or five alcoholic drinks nearly every day (36.5%) or from smoking one or more packs of cigarettes per day (35%). Further, a majority of youth (65.4%) did not perceive great risk from marijuana use once or twice a week [10].

Given the detrimental trends in and consequences of adolescent substance use, multi-level factors associated with adolescent substance use are well-documented. For example, Hawkins and colleagues [8] highlighted individual-level factors (e.g., sensation seeking, low harm avoidance, poor impulse control, and having problem behaviors), family-level factors (e.g., family conflict, parental and sibling alcoholism, and low bonding to family), peer-related factors (e.g., peer rejection and association with drug-using peers), and neighborhood-level factors (e.g., social disorganization and extreme economic deprivation). Brook and colleagues [7] also enumerated the primary risk factors for substance use ranging from individual demographic factors, such as gender, race/ethnicity, and employment to contextual factors, such as violence exposure, and neighborhood risks (e.g., lack of informal social controls, crime, and low-SES neighborhood).

In the etiology of adolescent substance use, limitations still remain. First, research on the determinants of adolescent substance use has focused primarily on individual, family, and peer factors [12,13,14]. Attention has recently shifted to examining the role of the surrounding environment in shaping substance using behaviors among youth [15,16,17,18,19]. Second, youth often encounter a constellation of risks simultaneously which makes them more vulnerable to negative developmental outcomes such as substance use initiation [20]. Thus, singular risk factors may be insufficient for predicting adolescent substance use [21]. In response, studies propose evaluating cumulative factors—the additive combination of multiple individual factors—for predicting adolescent substance use [20,21,22]. Third, it is well-known that early initiation of substance use is linked to heightened risk for substance abuse and dependence in later adulthood [23,24,25]. Accordingly, it is imperative to understand not only the factors related to adolescent substance use itself but also factors associated with the timing of substance use initiation. However, little is known about the factors related to the initiation of adolescent substance use partly because information for both the incidence and timing of substance use are not available in most data [18,20]. Fourth, noticeable increases in substance use initiation among ethnic minority youth have been found in recent years [10]. However, there is a paucity of research on substance use initiation of ethnic minority youth.

The current study addresses gaps in the literature on adolescent substance use by: (1) estimating the effects of cumulative exposure to neighborhood risk (i.e., neighborhood social disorder and neighborhood violent and property crime rates) and protective (i.e., neighborhood social capital) factors during preadolescence (ages 8–11) on the likelihood of initiating cigarette, alcohol, and marijuana use during adolescence (ages 12–18) among low-income Latinx and African American youth, after controlling for child, caregiver, and household factors; and (2) investigating whether these effects on substance use initiation varied by Latinx and African American race/ethnicity. In this study, we refer to Latinx and African American as racial/ethnic groups since both groups have considerable ethnic and racial mixing. According to the U.S. Office of Management and Budget, Latinx individuals may be of Cuban, Mexican, Puerto Rican, South or Central American, or other Spanish culture or origin regardless of race (https://www.census.gov/topics/population/hispanic-origin/about.html, accessed on 15 August 2021). African American is a social definition of race that is based upon self-identification and reflects individuals having origins in any of the racial groups in Africa. It is recognized that the race category in the United States includes racial, national origin or sociocultural groups (https://www.census.gov/topics/population/race/about.html, accessed on 15 August 2021). Findings will advance the understanding of the role of neighborhood effects on adolescent substance use initiation and may yield insights into community-level prevention and intervention programs for healthy development among economically disadvantaged ethnic minority groups.

## 2. Methods

### 2.1. Data and Sample

Data for this study were derived from the *Denver Child Study*, a retrospective study with current and former Denver Housing Authority (DHA) residents in Denver, Colorado [26,27]. It was conducted by an experienced research team that previously had worked with DHA residents on a series of program evaluation studies starting in 1994 that were independent from the Denver Housing Authority. The study was designed and implemented after several years of extensive fieldwork, considerable preliminary contact with residents and community stakeholders, and the completion of a feasibility study with residents in 1999–2001. Additionally, the research team included interviewers from racial and ethnic groups found in the resident population and multiple team members were able to conduct the interviews in Spanish. All interviewers completed 30 hours of training prior to conducting any interviews with study participants and were carefully supervised throughout the duration of the study. Caregivers were informed about the study team’s duty to warn about any instances of child abuse or neglect. Additionally, follow-up interviews conducted in 2010–2011 separately with a subset of caregivers and their older children (N = 83) to assess the quality of caregiver self-reports found considerable congruence (>90%) between caregiver and young adult reports of all child outcomes including substance use and violence victimization and perpetration.). The *Denver Child Study* was designed to assess the effects of neighborhood conditions on a wide variety of developmental outcomes of children and adolescents who resided a significant period of childhood in subsidized housing units operated by DHA and available to low-income households residing in the City and County of Denver (the Denver Housing Authority has 3000 subsidized units typically in row-type housing as well as 1500 dispersed housing units in single-family, duplexes, triplexes and fourplexes scattered throughout the City and County of Denver. Eligible families may earn between 0 to 120 of the area median income to qualify). The DHA used a quasi-random assignment strategy during the process of initial dwelling allocation that mimics random assignment of households to neighborhood characteristics, thereby reducing geographic selection bias associated with residential choice [28,29].

The *Denver Child Study* has two main data sources. First, between April 2006 and February 2008, initial interviews with primary caregivers, which included parents, grandparents, foster parents or other relatives who were responsible for the children in the household, collected a broad range of indicators of family and household characteristics as well as residential histories—all of which were verified using multiple techniques. All questions utilized in the *Denver Child Study* were either derived from previously validated instruments or were validated as part of the feasibility study conducted in 1999–2001 or our pretests of the final survey instrument in 2005 [26]. During the period from 2008 and 2012, *Denver Child Study* interview data were subjected to multiple rounds of verification including cross-checks with DHA administrative data and follow-up interviews with primary caregivers to address any noted discrepancies or omissions in survey responses. Further, residential history data also were verified using five different residential search engines; see details in authors [26,27]. All of these activities were completed before creating the child-year database of child, caregiver, household, and neighborhood characteristics utilized in this study. As noted in [27], while we acknowledge concerns about caregiver self-reports of these data, we found the incidence of caregiver-reported substance use by their adolescents was consistent to what has been reported for Colorado in other national surveys such as the National Survey on Drug Use and Health. The interviews were primarily completed by phone in either English or Spanish and lasted approximately 90 minutes. Data were collected for all eligible children associated with each household. Second, neighborhood indicators were added to the original survey data using household residential histories reported by primary caregivers. The source of indicators particularly relevant for this study came from the Denver-based *Piton Foundation’s Neighborhood Facts Database,* which compiled area-based administrative information for items such as violent crime rates and property crime rates per 1000 residents reported to police.

Participants in the *Denver Child Study* were restricted to current and former DHA residents who: first entered DHA in 1987 or later when the DHA began assignment to a dwelling unit from a common waiting list; had children aged 0 and 18 years in their home when they moved into these DHA units; had resided in DHA for at least two years before their children reached age 19 in order to be exposed to neighborhood conditions for a sufficient period of time; and were of either Latinx or African American ethnicity. The initial sample in the *Denver Child Study* consisted of 710 households and their 1702 Latinx and African American children and adolescents. Of these 1702 children and adolescents, participants in this study were restricted to: (1) children aged 12 or older at the time of survey; (2) who initiated substance use after they moved into their initial DHA unit and who first started their use of a specific substance after they reached the age of 12; and (3) adolescents who initiated the use of only one type of substance between the ages of 12 and 18 The final analysis sample for this study included 736 adolescents residing in 400 different households. The protocol for this secondary data analysis was reviewed and exempted by the first author’s Institutional Review Board (IRB-2015-1224).

### 2.2. Measures

#### 2.2.1. Adolescent Substance Use Initiation

Initiation of adolescent substance use in this study was defined using caregiver reports of the first initiation of cigarette, alcohol, and marijuana use while they lived in DHA housing with their eligible children and when these children were between the ages of 12 and 18 years [26]. Primary caregivers were asked: “When your child(ren) lived with you in DHA housing and they were under age 18, did any of your children ever smoked tobacco [ever drank alcohol, ever used marijuana (pot)]?” These three variables were measured dichotomously 0 = never smoked tobacco, never drank alcohol, or never smoked marijuana, respectively or 1 = ever smoked tobacco, ever drank alcohol, and ever smoked marijuana, respectively.

Primary caregivers who responded “yes” to the above questions of experiences of substance use were then asked, “How old was your child when he/she first began to smoke cigarettes [drink alcohol, smoke marijuana]?” Age at the initiation of each substance was coded in years. For youth who did not have any experience of substance use, their age of the initiation was coded as either 18 years if over 18 or the actual age at time of survey if the youth was younger than 18. The three variables of adolescent experiences of cigarette, alcohol, and marijuana use were then combined into an overall categorical measure of substance use initiation. Adolescents who did not initiate any of the three substances represent no substance initiation (coded as 0). The remaining categories of the outcome measure of the initiation of substance use represent cigarette use (coded as 1), alcohol use (coded as 2), and marijuana use (coded as 3). The primary focus of this study is to investigate the effects of neighborhood characteristics on the overall model where all three types of substance use initiation are treated alike as well as the cause specific model which assesses the effects on each type of substance use initiation. As a result, the outcome variable is a categorical variable with four mutually exclusive categories. Therefore, those who initiated multiple substances during adolescence had to be excluded.

#### 2.2.2. Neighborhood Social Disorder

Neighborhood social disorder was defined using primary caregivers’ perceptions of the presence of dangerous conditions in the neighborhood. The *Denver Child Study* asked caregivers to recall conditions in their neighborhood, “Tell me whether your neighborhood had the following negative neighborhood conditions: people selling drugs; gang activity; homes broken into by burglars; people being robbed or mugged often; and people getting beaten or raped” [26]. Response options for each question are either yes (coded as 1) or no (coded as 0). Next, the *Denver Child Study* estimated whether children lived in neighborhoods with any of these conditions during each child year between the ages of 8 and 11. Accordingly, the total number of years residing in a neighborhood with one or more of these conditions was summed and ranged from 0 to 4 years.

#### 2.2.3. Neighborhood Violent and Property Crime Rates

Two indicators for neighborhood crime: violent crime rates and property crime rates per 1000 populations were derived from administrative records reported to police and available from the *Piton Neighborhood Facts* database [26]. Information about violent and property crime rates were added to the original survey data using household residential histories for each child year. This study then calculated neighborhood averages for violent crime rates and property crime rates between the ages of 8 and 11.

#### 2.2.4. Neighborhood Social Capital

Social capital was defined as social connectedness and involvement in local organizations that inclines individuals to do things for each other. The *Denver Child Study* asked caregivers to recall conditions in each of the residential locations they resided over the course of their children’s childhood: “Tell me whether your neighborhood had the following: people who could get together to solve neighborhood problems; many neighbors who watch out for my children and property; many neighbors who knew me and my children by name; many adult neighbors you and your children could look up to; and many neighbors you could count on for help in times of trouble.” In addition, caregivers were asked if they were active in any organizations such as block clubs, tenant groups, or religious organizations located in each of these neighborhood locations [26]. For each of these indicators, the response categories were either yes (coded as 1) or no (coded as 0) indicating the presence or absence of the indicator during a given child year. In this study, the total years of residing neighborhood with one or more of the five characteristics between the ages of 8 and 11 ranged from 0 to 4 years, respectively.

#### 2.2.5. Covariates

Child, caregiver, and household factors were selected based on the extant literature on adolescent substance use and included in this study as covariates. Gender (Male = 1), race/ethnicity (African American = 1, Latino = 0), violence victimization, and parent-child relationships are represented in the child risk factor. Violence victimization in this study was defined as children’s experiences of violence in or around their school or neighborhood. It was measured as total number of years that a child was a victim of school or neighborhood violence between the ages of 8 and 11. The types of violence in the data include being beaten up, chased, or robbed. Parent-child relationships in this study were measured by asking caregivers to rate the influence that each biological parent had on their children during childhood using a four-point Likert scale ranging from very negative (=0) to very positive (=4). The two items were then summed into an overall measure of the parent-child relationship. The score ranged from a low of 0 to a high of 8; higher scores represent more positive relationships between children and their biological parents during childhood. The caregiver risk factor included caregiver educational attainment (less than high school degree = 1), caregiver earnings (less than $8549 = 1), single parenthood (yes = 1), and caregiver history of substance use (ever used substance = 1). Household stressors and residential mobility were used to create the household risk factor (see Table 1). Household stressors reflect the total number of years that households experienced one or more of five stressful events—difficulty in paying bills, major illness or injury, insufficient money for food, utility shutoffs, and eviction—between the ages of 8 and 11 with a range of 0 to 4 years. Residential mobility refers to the total number of moves between initial DHA assignment and the initiation of substance use. For those who did not use substances, these moves were estimated through age 18 or age at the time of survey if the youth was younger than 18.

### 2.3. Creation of Cumulative Factors

In order to create cumulative risk factors, all indicators in each domain were dichotomized reflecting either presence (coded as 1) or absence of the risk or protection domain (coded as 0). For continuous indicators in each domain, cutoff points were set to either the bottom 25th or the top 75th percentile values similar to procedures for formulating cumulative risk factors that have been used in previous studies [22,30]. For caregiver earnings, the median value of $8549 was used as the cutoff point.

### 2.4. Data Analysis

Prior to conducting any substantive analyses, descriptive statistics were examined for initial indicators across different domains. This study utilized the Cox cause-specific hazards model with robust standard errors to examine the cumulative effects of neighborhood risk and protective factors during preadolescence on the likelihood of the initiation of cigarette, alcohol, and marijuana use during adolescence after controlling for race/ethnicity as well as child, caregiver, and household risk factors. The Cox cause-specific hazards model is an appropriate statistical method to assess the occurrence and timing of events, particularly when each participant in the data can experience only one of several different types of event [31,32,33]. Preliminary data analyses were conducted using SPSS statistical software (version 27, IBM^®^SPSS Statistics, Chicago, IL, USA) and the analyses of primary multivariate models were conducted using STATA (version 14, StataCorp, College Station, TX, USA).

## 3. Results

### 3.1. Descriptive Statistics

Table 1 presents descriptive information for the full sample (N = 736) and by race/ethnicity. The majority of the sample (53%) were of Latinx ethnicity. Almost half of the sample (49%) was male. Nearly 98% of adolescents in the study had not been victims of violence in their neighborhoods or schools during preadolescence; however, 2% experienced one or more years of violence victimization. The parent–child relationship score ranged from 0 to 8 with the mean score of 5.8 for this sample indicating a more positive relationship. Over half (52%) of the caregivers did not hold a high school degree. Average annual earnings were $11,140 (*SD* = $11,087) with the median value of $8549. Nearly 64% of the caregivers were single parents during the preadolescence period. Moreover, 14% of caregivers reported they had a history of substance use since becoming parents. Households experienced various financial and physical hardships, including difficulty in paying bills (Mean = 1.6 years, *SD* = 1.8), illness and injuries (Mean = 0.9 years, *SD* = 1.5), insufficient money for food (Mean = 1.6 years, *SD* = 1.8), utility shutoffs (Mean = 0.8 years, *SD* = 1.4), and evictions (Mean = 0.2 years, *SD* = 0.7). On average, these households had moved two times (*SD* = 1.5) between their initial DHA assignment and their child’s initiation of substance use.

In terms of neighborhood social disorder, the average number of years during preadolescence that children lived in neighborhoods with drug activities, gangs, burglaries, robberies, and people getting beaten or raped were 1.9 (*SD* = 1.8), 2.0 (*SD* = 1.8), 1.6 (*SD* = 1.8), 1.2 (*SD* = 1.6), and 1.2 (*SD* = 1.6), respectively. The average violent crime rate and property crime rate per 1000 residents for the period was 13.2 (*SD* = 8.6) and 75.6 (*SD* = 47.9), respectively. Youth resided in neighborhoods with varying degrees of social capital during preadolescence. Exposure to different forms of neighborhood social capital ranged from 1.3 years (*SD* = 1.7) for being active in an organization in the neighborhood to 3.3 years (*SD* = 1.4) for the presence of neighbors who knew caregivers and their children by name.

### 3.2. Prevalence of and Age at Substance Use Initiation

Table 2 presents the prevalence and average age of initiation of substance use. Approximately 84% of the adolescents in our study did not initiate any substance use when they were between the ages of 12 and 18 years. Among adolescents who initiated one of the three types of substance use, 5.6% initiated cigarette use, 5.4% initiated alcohol use, and 5.2% used marijuana for the first time during adolescence. On average, adolescents in this study initiated their use of cigarettes, alcohol, and marijuana when they were 16.0 (*SD* = 1.6), 16.0 (*SD* = 1.4), and 15.7 (*SD* = 1.8) years old, respectively. Latinx youth, on average, initiated their use of cigarettes (15.8, *SD* = 1.6) and alcohol (15.6, *SD* = 1.3) earlier than African American youth (16.3, *SD* = 1.6, and 16.0, *SD* = 1.4, respectively). Average age of the initiation of marijuana use for both groups was identical (i.e., 15.7 years).

### 3.3. Effects of Cumulative Exposure to Neighborhood Conditions and Substance Use Initiation

Table 3 presents the results of the Cox cause-specific hazard model with robust standard errors, which are adjusted for the clustering of the data by household. In the overall model where all three types of the initiation of substance use are treated alike, residing in neighborhoods with higher levels of social disorder significantly increased the likelihood of the initiation of substance use. As adolescents were exposed to increased levels of neighborhood social disorder, their hazard for the initiation of substance use in general increased by 25% (95% CI = 1.083–1.433, *p* = 0.002). In addition, greater exposure to neighborhood social disorder during preadolescence was associated with a 36% (95% CI = 1.062–1.745, *p* = 0.015) higher hazard of initiation of cigarette use during adolescence.

### 3.4. Ethnic Differences in the Effects of Cumulative Exposure to Neighborhood Conditions on Substance Use Initiation

Table 4 presents the results of the stratified models by race/ethnicity. In the overall model for African American youth, neighborhood social disorder during preadolescence significantly increased the likelihood of the initiation of substance use during adolescence in general. Specifically, greater exposure to neighborhood social disorder during preadolescence was associated with a 36% (95% CI = 1.125–1.637, *p* = 0.001). Higher hazard of the initiation of substance use during adolescence among African American youth (see Panel A). For Latinx youth, exposure to higher levels of neighborhood social disorder during preadolescence increased the hazard of initiating cigarette use during adolescence among Latinx adolescents by 42% (95% CI = 1.031–1.966, *p* = 0.032) (see Panel B).

### 3.5. Sensitivity Tests to Check the Robustness of Findings

To check the robustness of our findings, we ran sensitivity analyses that included a sample of cases of youth with multiple substance use initiation to examine: (1) cumulative substance use initiation with categories ranging from no substance use initiation (reference category) to use of up to three substances during adolescence; and (2) the type of substance use initiation with four categories (i.e., cigarette, alcohol, marijuana, and polysubstance use). Following conventions in the literature, polysubstance use is defined as initiation of two or more of the three substances we examined in the study. Using these two newly created outcomes, we ran Cox cause-specific hazard models to test whether the neighborhood effects noted above were notably different with the inclusion of this subset of youth. These additional analyses are available in the online Supplementary Materials. Below, we summarize the results for neighborhood risk and protective factors only.

As shown in online Supplementary Materials Table S1, neighborhood social disorder remains a significant risk factor for ever initiating substance use (HR = 1.204, 95% CI = 0.092, 1.326, *p* = 0.000). Moreover, when cumulative substance use initiation was used as the outcome variable, the hazard ratios for neighborhood social disorder were 1.273 (95% CI = 1.115, 1.452, *p* = 0.000) and 1.220 (95% CI = 1.034, 1.441, *p* = 0.019) for the initiation of one and two substances, respectively. However, it was not a significant predictor for the initiation of three or more substances. Neither neighborhood crime rates nor neighborhood social capital predicted cumulative substance use initiation. In the stratified model by race/ethnicity presented in Supplementary Materials Table S2, the hazard ratios for neighborhood social disorder for African American adolescents were 1.229 (95% CI = 1.073, 1.408, *p* = 0.003 for ever initiating substance use and 1.396 (95% CI = 1.183, 1.648, *p* = 0.000) for initiation of one substance but not for the initiation of two or three substances. For Latinx adolescents, the hazard ratio for neighborhood social disorder was significant only as a predictor of the initiation of two substances during adolescence (HR = 1.224, 95% CI = 1.008, 1.486, *p* = 0.041). Additionally, neighborhood violent and property crime rates lowered the hazard of initiating two substances (HR = 0.598, 95% CI = 0.383, 0.936, *p* = 0.025) for Latinx youth only but did not affect the initiation of only one or all three substances during adolescence.

As reported in Supplementary Materials Table S3, when we analyzed initiation by the type of substance and included polysubstance use as one of the possibilities in the outcome variable, the hazard ratios for neighborhood social disorder were 1.204 (95% CI = 1.092, 1.326, *p* = 0.000) for ever initiating any use during adolescence, and 1.184 (95% CI = 1.002, 1.400, *p* = 0.047) and 1.252 (95% CI = 1.025, 1.529, *p* = 0.028) for the initiation of cigarette and alcohol use, respectively. However, neighborhood crime rates and neighborhood social capital did not significantly affect the initiation of cigarette or alcohol use. Furthermore, there were no significant neighborhood effects on initiating marijuana use or polysubstance use during adolescence for the full sample. When we stratified the samples by race/ethnicity (see Supplementary Materials Table S4), neighborhood social disorder was a significant factor only for cigarette use initiation by American youth with a hazard ratio of 1.428 (95% CI = 1.143, 1.784, *p* = 0.002). For Latinx adolescents, however, the hazard ratios for neighborhood social disorder were 1.508 (95% CI = 1.141, 1.994, *p* = 0.004) and 1.263 (95% CI = 1.002, 1.521, *p* = 0.048) for alcohol and polysubstance use, respectively, but did not influence cigarette or marijuana use. Additionally, neighborhood violent and property crime rates lowered the hazard of initiating alcohol use by Latinx youth (HR = 0.604, 95% CI = 0.387, 0.941, *p* = 0.026) but not the initiation of the other substances. Neither did neighborhood violent and property crime rates significantly influence the type of substance use initiated by African American youth. Finally, neighborhood social capital was not found to be a significant factor mitigating substance use initiation by either African American or Latinx adolescents.

The results of the sensitivity analyses indicate that the effects of cumulative neighborhood conditions vary slightly across cumulative substance use initiation as well as the types of substance with polysubstance. Nonetheless, our overall conclusion based upon these sensitivity analyses is that the results are quite similar to the findings presented in Table 3 and Table 4. Cumulative substance use initiation and polysubstance use, however, are suggested avenues for future research since the longitudinal data needed to fully explore such use are not available in the *Denver Child Study* dataset.

## 4. Discussion

Despite the gradual decline in prevalence rates of adolescent substance use by American youth in recent years, the initiation of such use has remained a topic of concern for health and behavioral scientists because of its link to harmful developmental outcomes as well as increased mortality and morbidity [7,8]. Of particular concern is the association between the initiation or experimentation of substance use during adolescence and substance abuse and dependence in later adulthood [23,24,25]. This study contributes to the literature by estimating the cumulative effects of exposure to neighborhood contexts during preadolescence on the initiation of substance use during adolescence by Latinx and African American youth who resided in randomly assigned public housing units and neighborhoods in Denver, Colorado. The results drawn from this study suggest that exposure to neighborhood social disorder characterized by drug dealing, gang activities, robberies, and assault within communities is a significant risk factor, especially for the initiation of cigarette use. Previous studies (see summary in [27]), have produced mixed findings regarding the exposure to neighborhood social disorder on alcohol or marijuana consumption including heightened use in more affluent neighborhoods where youth are more likely to have access to or afford these substances; the presence of family support networks that moderate such use; increased geographic restrictions on activity spaces by Latinx and African American parents who may invoke more intensive monitoring of their youth in neighborhoods perceived as more dangerous; and cost-benefit assessments of substance use made by youth because of heightened police surveillance targeting disadvantaged neighborhoods.

The significant effect of neighborhood social disorder on the initiation of adolescent substance use is consistent with previous studies [18,34,35,36,37]. For example, Wilson and colleagues [37] reported significant positive relationships between neighborhood disorder and alcohol, tobacco, and marijuana use among middle school students. Burlew and colleagues [35] also found that neighborhood disorder was a significant predictor of initiating cigarette, alcohol, and marijuana use among African American youth. A longitudinal latent transition analysis by Reboussin and colleagues [18] also reported similar results linking increased neighborhood disorder with increased use of marijuana among African American adolescents.

One salient explanation for the relationship between neighborhood social disorder and substance use initiation may be that the visual cues of neighborhood social disorder may affect residents’ perceptions of their neighborhoods as dangerous, threatening, or stressful. Consequently, adolescents might use substances as a coping mechanism in light of these risks and other tangible stressors they may face living in precarious households [36]. Community norms in disordered neighborhoods also may play a role in substance using behaviors among adolescents: the increased visibility of substance use by peers, family members, or adults within communities may increase the initiation of substance use among youth [38,39].

The findings of this study support racial/ethnic differences in the relationship between neighborhood social disorder and substance use initiation. The effect of neighborhood social disorder during preadolescence on the initiation of cigarette use during adolescence was significant only for Latinx youth in the stratified model. Previous literature suggests that increased tobacco use among Latinx youth may be associated with acculturative stress, the erosion of social networks and family ties, as well as the extensive media advertising and promotion campaigns targeting disadvantaged Latinx neighborhoods [38,40].

### 4.1. Strengths, Limitations, and Future Directions

This study has several strengths. First, this study utilized data that significantly ameliorated geographic selection bias through quasi-random assignment to neighborhood, thereby providing plausible causal associations between neighborhood conditions and the initiation of adolescent substance use [26]. This study also incorporated a substantial number of contextual factors (i.e., total 27 indicators) across child, caregiver, household, and neighborhood. In addition, this study focused on the cumulative effects of these contextual factors during preadolescence on the initiation of adolescent substance use [22]. This study also contributes to the current knowledge of adolescent substance use initiation by addressing the role of neighborhood conditions, particularly neighborhood social disorder and neighborhood social capital, which have been previously understudied. In particular, the results underscore the importance of understanding how neighborhood social disorder is associated with the initiation of adolescent substance use.

This study also has several limitations that should be acknowledged. First, Latinx and African American youth in the study came from low-income households residing in subsidized housing. Accordingly, the results of this study are not generalizable to all youth. Future research should be conducted with other samples of adolescents, preferably using natural or quasi-random experiments, to enhance the generalizability of the findings as well as ameliorate geographic selection bias. Second, the data for this study were derived from a retrospective survey with primary caregivers. The occurrence and timing of the initiation of substance use as well as perceptions about neighborhood conditions are based on primary caregivers’ self-reports, which may be subject to recall as well as response bias. Thus, future research needs to include reports from adolescents themselves in order to estimate more accurate rates of initiation of substance use as well as their perceptions about neighborhood influences. Third, this study utilized cumulative factors in explaining the initiation of adolescent substance use. Although the use of cumulative risk factors in the prediction of adolescent substance use might be theoretically relevant and empirically beneficial, the approach of using the bottom or top quartile as cutoff points for determining a variable as a risk or protective factor might have either underestimated or overestimated the level of risk or protection for a particular factor. Fourth, although this study controlled for a number of potential confounders of child, caregiver, and household factors, there is the possibility of unmeasured factors that might affect the initiation of adolescent substance use (e.g., physical availability of substances within communities).

### 4.2. Implications for Practice and Policy

Substance use is one of the serious health risks faced by American adolescents. Various intervention and prevention approaches at the individual- and family-level have been implemented to reduce substance use among adolescents such as cognitive and behavioral therapy and family systems approaches [41,42]. Individual-level interventions have mainly focused on changing unhealthy patterns of thinking and beliefs that might contribute to the initiation of substance use among adolescents [43]. Family-level interventions have addressed the characteristics of interactions within families (e.g., poor communications and family bonding) which might encourage adolescent substance use initiation [41]. Although these individual- and family-level interventions may yield significant reductions in substance use among adolescents, they do not consider how social dimensions of neighborhood as broader contexts promulgate or mitigate adolescent substance use. The results from this study suggest that differences in levels of exposure to neighborhood social disorder during preadolescence may have a significant effect on substance use initiation during adolescence even after controlling for individual, caregiver, and household risk factors. Therefore, effective intervention and prevention strategies need to consider the social dimensions of neighborhood disorder (e.g., the presence of gangs, drug dealing, and violence) in order to reduce the stressors that may be associated with the initiation of adolescent substance use.

The results of this study also inform policies addressing adolescent substance use. First, this study’s findings suggest the importance of the role that neighborhood disorder might play in relation to adolescent substance use initiation. Thus, safety issues found disproportionately in disordered neighborhoods, such as heightened risk of victimization, might be addressed through implementation of youth programming in safe spaces such as community or neighborhood recreation centers. Additionally, community-led efforts such as neighborhood watch groups where parents and other adult role models serve to monitor local youth and enforce norms of acceptable behavior need to be encouraged. Such neighborhoods can be further enhanced by block clubs that create and maintain new gathering spaces for use by all community members (i.e., pocket parks, community gardens). Specifically, policies and programs aimed at enhancing alternative activities available to youth or to promote community collective efficacy and engagement in such neighborhoods may reduce drug dealing, gang activities, and violence, which, in turn, may lead to decreases in substance use among adolescents. Offering youth alternative spaces in which to interact, such as community or recreation centers and programming that promotes healthy activities and lifestyles, could mitigate safety concerns and alleviate the stressors that might trigger substance use. Finally, given the complexities of the role that risk and protective factors play in the prediction of substance use initiation across race/ethnicity and different types of substances, evidence-based intervention and prevention programs need to attend to the ways in which these multiple dimensions are addressed in more customized or tailored ways.

## 5. Conclusions

This study contributes to current knowledge of adolescent substance use by estimating the cumulative effects of exposure to neighborhood contexts during preadolescence on the initiation of substance use during adolescence by Latinx and African American adolescents who resided in randomly assigned subsidized housing units in Denver, Colorado, reported by their primary caregivers. The results drawn from this study suggest that exposure to neighborhood social disorder during preadolescence is a significant risk factor, especially for the initiation of cigarette use among adolescents. Additionally, the effect of preadolescent exposure to neighborhood social disorder on the initiation of cigarette use varies by Latinx and African American youth. These findings suggest the need for further research on the links between exposure to neighborhood social disorder and adolescent substance use initiation in order to develop and implement community-based prevention and intervention programs that reduce substance use initiation and facilitate healthy adolescent development.

## Figures and Tables

**Table 1:** Descriptive statistics for full sample and by Latinx and African American ethnicity.

Indicator	Overall (N = 736)		African American (N = 348)		Latino (N = 388)	
	%/Mean	*SD*	%/Mean	*SD*	%/Mean	*SD*
Race/Ethnicity (African American = 1)	47.3	0.5				
**Indicators for Child Risk Factor**						
Gender (Male = 1)	48.8	0.5	48.0	0.5	49.5	0.5
Violence Victimization (# of years)	0.2	0.7	0.1	0.6	0.2	0.7
Parent-child relationship (range = 0–8)	5.8	1.6	5.7	1.6	5.8	1.6
**Indicators for Caregiver Risk Factor**						
Caregiver educational attainment (No high school degree = 1)	51.9	0.5	54.9	0.5	49.2	0.5
Caregiver earnings ($)	11,139.6	11,087.3	11,248.8	10,977.4	11,041.6	11,198.2
Single parenthood (%)	63.9	43.8	59.7	44.8	67.6	42.5
Caregiver history of substance use (Yes = 1)	13.7	0.3	14.9	0.4	12.6	0.3
**Indicators for Household Risk Factor**						
Had difficulty in pay bills	1.6	1.8	2.0	1.8	1.3	1.7
Had major illness or injuries	0.9	1.5	1.0	1.6	0.9	1.5
Had not enough money for food	1.6	1.8	1.9	1.9	1.2	1.7
Had electricity, gas, or phone service cut off	0.8	1.4	1.0	1.5	0.7	1.4
Got evicted	0.2	0.7	0.3	0.8	0.1	0.5
Residential mobility (# of moves between the initial assignment and initiation of substance use)	2.2	1.5	2.2	1.6	2.2	1.5
**Indicators for Neighborhood Risk and Protective Factors**						
**Neighborhood social disorder** (# of years present in neighborhood)						
Selling drugs	1.9	1.8	1.9	1.8	1.8	1.8
Gangs	2.0	1.8	2.0	1.8	2.0	1.8
Burglaries	1.6	1.8	1.7	1.8	1.5	1.7
Robberies	1.2	1.6	1.2	1.7	1.1	1.6
People getting beaten or raped	1.2	1.6	1.2	1.6	1.2	1.6
**Neighborhood violent and property crime rates**						
Violent crime rates (per 1000 residents)	13.2	8.6	13.3	8.7	13.2	8.5
Property crime rates (per 1000 residents)	75.6	47.9	75.0	44.8	76.2	50.5
**Neighborhood social capital** (# of years present in neighborhood)						
People who could get together to solve neighborhood problems	2.2	1.8	2.1	1.8	2.3	1.8
Neighbors who watch out for children and property	2.7	1.7	2.6	1.7	2.8	1.6
Neighbors who knew primary caregiver and children by name	3.3	1.4	3.3	1.4	3.3	1.3
Adult neighbors primary caregiver and children could look up to	2.3	1.8	2.1	1.8	2.4	1.8
Neighbors you could count on for help in times of trouble	2.7	1.7	2.5	1.8	2.9	1.6
Being active in organizations in neighborhood	1.3	1.7	1.2	1.7	1.3	1.7

All time-varying indicators (i.e., violence victimization, single parenthood, caregiver earnings, all indicators for household risk factors, and all indicators for neighborhood factors) were measured when the child was between the ages of 8 to 11. Risk and protective factors are bolded.

**Table 2:** Prevalence and average age of substance use initiation.

Substance Use Initiation	Overall (N = 736)		African American (N = 348)		Latinx (N = 388)	
	N (%)	*SD*	N (%)	*SD*	N (%)	*SD*
None	617 (83.8)	2.3	294 (84.5)	2.2	323 (83.2)	2.3
Cigarette Use	41 (5.6)	0.2	17 (4.9)	0.2	24 (6.2)	0.2
Age of Cigarette Use Initiation (Mean)	16.0	1.6	16.3	1.6	15.8	1.6
Alcohol Use	40 (5.4)	0.2	20 (5.7)	0.2	20 (5.2)	0.2
Age of Alcohol Use Initiation (Mean)	16.0	1.4	16.0	1.4	15.6	1.3
Marijuana Use	38 (5.2)	0.2	17 (4.9)	0.2	21 (5.4)	0.2
Age of Marijuana Use Initiation (Mean)	15.7	1.8	15.7	1.8	15.7	1.9

**Table 3:** Cox cause-specific hazards model regressing race/ethnicity, child, caregiver, household, and neighborhood risk factors on substance use initiation.

	Overall		Cigarette		Alcohol		Marijuana	
Risk Factor	HR (95% CI)	*p* Value	HR (95% CI)	*p* Value	HR (95% CI)	*p* Value	HR (95% CI)	*p* Value
Race/Ethnicity (African American = 1)	0.937 (0.640–1.371)	0.737	0.714 (0.359–1.420)	0.337	1.166 (0.584–2.328)	0.663	0.989 (0.500–1.955)	0.975
Child Risk Factor	1.220 (0.939–1.586)	0.136	0.967 (0.605–1.547)	0.890	1.305 (0.843–2.021)	0.232	1.456 (0.908–2.334)	0.119
Caregiver Risk Factor	1.203 (0.974–1.486)	0.087	1.279 (0.925–1.767)	0.137	1.313 (0.914–1.885)	0.140	1.035 (0.755–1.420)	0.831
Household Risk Factor	0.979 (0.794–1.207)	0.844	1.159 (0.815–1.649)	0.410	0.928 (0.587–1.468)	0.750	0.858 (0.642–1.147)	0.302
Neighborhood Risk Factors								
Neighborhood Social Disorder	**1.246** (1.083–1.433)	0.002	**1.362** (1.062–1.745)	0.015	1.177 (0.896–1.545)	0.242	1.179 (0.962–1.445)	0.113
Neighborhood Violent and Property Crime Rates	0.910 (0.701–1.182)	0.480	0.798 (0.508–1.251)	0.324	0.990 (0.645–1.519)	0.962	0.958 (0.566–1.623)	0.874
Neighborhood Social Capital	0.985 (0.891–1.090)	0.772	1.000 (0.837–1.194)	0.997	0.931 (0.775–1.119)	0.448	1.026 (0.862–1.221)	0.772

Unstandardized exponentiated coefficients (HR = hazard ratio) are presented in the first column. CI = confidence interval. Significant hazard ratios are bolded.

**Table 4:** Results of stratified Cox cause-specific hazards model.

Panel A. African American Youth (N = 348)								
	Ever Initiated		Cigarette Use		Alcohol Use		Marijuana Use	
Risk Factor	HR (95% CI)	*p* Value	HR (95% CI)	*p* Value	HR (95% CI)	*p* Value	HR (95% CI)	*p* Value
Child risk factor	1.333 (0.896–1.982)	0.156	1.669 (0.956–2.913)	0.072	1.164 (0.574–2.362)	0.673	1.298 (0.583–2.890)	0.523
Caregiver risk factor	1.355 (0.995–1.845)	0.054	1.527 (0.902–2.585)	0.115	1.626 (0.988–2.676)	0.056	1.052 (0.530–2.088)	0.884
Household risk factor	0.917 (0.706–1.191)	0.515	**1.611** (1.103–2.354)	0.014	0.813 (0.502–1.318)	0.402	**0.554** (0.355–0.864)	0.009
Neighborhood Risk Factors								
Neighborhood social disorder	**1.357** (1.125–1.637)	0.001	1.344 (0.928–1.945)	0.118	1.329 (0.969–1.824)	0.078	1.353 (0.984–1.860)	0.063
Neighborhood violent and property crime rates	1.100 (0.774–1.565)	0.594	0.722 (0.366–1.425)	0.348	1.402 (0.832–2.362)	0.204	1.241 (0.617–2.496)	0.545
Neighborhood social capital	0.911 (0.785–1.057)	0.217	0.87 (0.654–1.156)	0.336	0.901 (0.698–1.163)	0.422	0.954 (0.703–1.296)	0.765
**Panel B. Latinx Youth (N = 388)**								
	**Ever Initiated**		**Cigarette Use**		**Alcohol Use**		**Marijuana Use**	
**Risk Factor**	**HR (95% CI)**	***p* Value**	**HR (95% CI)**	***p* Value**	**HR (95% CI)**	***p* Value**	**HR (95% CI)**	***p* Value**
Child risk factor	1.178 (0.851–1.632)	0.323	0.677 (0.383–1.197)	0.180	1.509 (0.895–2.544)	0.122	**1.697** (1.006–2.863)	0.047
Caregiver risk factor	1.151 (0.891–1.487)	0.282	1.145 (0.744–1.761)	0.539	1.194 (0.743–1.919)	0.463	1.109 (0.835–1.474)	0.475
Household risk factor	1.097 (0.810–1.484)	0.550	0.847 (0.488–1.471)	0.556	1.170 (0.574–2.388)	0.665	1.384 (0.949–2.019)	0.091
Neighborhood Risk Factors								
Neighborhood social disorder	1.139 (0.949–1.368)	0.163	**1.424** (1.031–1.966)	0.032	0.986 (0.674–1.442)	0.943	0.988 (0.807–1.209)	0.905
Neighborhood violent and property crime rates	0.758 (0.546–1.051)	0.096	0.907 (0.521–1.580)	0.731	0.644 (0.361–1.150)	0.137	0.713 (0.416–1.224)	0.220
Neighborhood social capital	1.071 (0.934–1.228)	0.324	1.044 (0.824–1.323)	0.721	1.002 (0.773–1.297)	0.990	1.163 (0.957–1.413)	0.129

Unstandardized exponentiated coefficients (HR = hazard ratio) are presented in the first column. CI = confidence interval. Significant hazard ratios are bolded.

## Data Availability

The data for this study are under a Data Confidentiality Agreement with the Denver Housing Authority. Requests for the data analyses supporting the research in this study may be made to the first author.

## Supplementary Materials

The following are available online at https://www.mdpi.com/article/10.3390/ijerph182010831/s1, Table S1: Substance Use Initiation during Adolescence by Number of Substances (N = 946); Table S2: Substance Use Initiation during Adolescence by Race/Ethnicity and Number of Substances; Table S3: Substance Use Initiation during Adolescence by Type of Substance (N = 946); Table S4: Substance Use Initiation during Adolescence by Race, Ethnicity and Type of Substance.
