# Supplementary material for: Cumulative Exposure to Neighborhood Conditions and Substance Use Initiation among Low-Income Latinx and African American Adolescents
Source: Int J Environ Res Public Health. 2021 Oct 15;18(20):10831. doi: 10.3390/ijerph182010831 (PMC8535668; doi:10.3390/ijerph182010831)
Supplement: Supplementary file 1 [file ijerph-18-10831-s001.zip › Supplementary Materials.pdf]

Table S1. Substance Use Initiation during Adolescence by Number of Substances (n=946).

	**Ever Initiated Substance Use**		**One Substance**		**Two Substances**		**Three Substances**	
	**HR (95% CI)**	**P Value**	**HR (95% CI)**	**P Value**	**HR (95% CI)**	**P Value**	**HR (95% CI)**	**P Value**
Race/Ethnicity (African American = 1)	0.852 (0.609,1.191)	0.348	1.065 (0.701, 1.618)	0.767	0.572 (0.311, 1.052)	0.072	0.775 (0.383, 1.568)	0.478
Child Risk Factor	**1.339** (1.105,1.622)	0.003	1.156 (0.893, 1.498)	0.272	**1.505** (1.040, 2.178)	0.030	**1.655** (1.170, 2.342)	0.004
Caregiver Risk Factor	1.066 (0.896,1.267)	0.472	1.225 (0.996, 1.506)	0.054	1.179 (0.872, 1.593)	0.284	**0.675** (0.498, 0.913)	0.011
Household Risk Factor	**0.739** (0.559,0.976)	0.033	**0.699** (0.503, 0.971)	0.033	0.660 (0.370, 1.177)	0.160	0.996 (0.525, 1.890)	0.991
Neighborhood Risk Factors								
Neighborhood Social Disorder	**1.204** (1.092,1.326)	0.000	**1.273** (1.115, 1.452)	0.000	**1.220** (1.034, 1.441)	0.019	1.004 (0.828, 1.217)	0.968
Neighborhood Violent and Property Crime Rates	0.872 (0.722,1.053)	0.155	0.819 (0.630, 1.065)	0.136	0.868 (0.590, 1.276)	0.471	1.064 (0.737, 1.536)	0.739

Neighborhood Social Capital	0.946 (0.877,1.020)	0.15	0.986 (0.892, 1.090)	0.784	0.863 (0.736, 1.012	0.070	0.932 (0.806, 1.078)	0.342

Unstandardized exponentiated coefficients (HR = Hazard Ratio) are presented in the first column. CI = Confidence Interval. Significant hazard ratios are bolded.

Table S2. Substance Use Initiation during Adolescence by Race/Ethnicity and Number of Substances.

**Panel A. African American Youth (n=436)**	**Ever Initiated Substance Use**		**One Substance**		**Two Substances**		**Three Substances**	
	**HR (95% CI)**	**P Value**	**HR (95% CI)**	**P Value**	**HR (95% CI)**	**P Value**	**HR (95% CI)**	**P Value**
Child Risk Factor	1.269 (0.963, 1.673)	0.091	1.140 (0.779, 1.669)	0.500	1.502 (0.966, 2.334)	0.071	**1.597** (1.052, 2.424)	0.028
Caregiver Risk Factor	0.977 (0.752, 1.270)	0.862	1.247 (0.941, 1.652)	0.124	0.996 (0.589, 1.683)	0.987	**0.427** (0.228, 0.798)	0.008
Household Risk Factor	0.626 (0.442, 0.887)	0.008	**0.611** (0.407, 0.918)	0.018	0.468 (0.210, 1.045)	0.064	0.939 (0.348, 2.536)	0.902
Neighborhood Risk Factors								
Neighborhood Social Disorder	**1.229** (1.073, 1.408)	0.003	**1.396** (1.183, 1.648)	0.000	1.196 (0.874, 1.637)	0.264	0.803 (0.576, 1.120)	0.196
Neighborhood Violent and Property Crime Rates	0.996 (0.746, 1.330)	0.980	0.845 (0.563, 1.268)	0.416	1.502 (0.859, 2.627)	0.154	1.241 (0.658, 2.342)	0.505
Neighborhood Social Capital	0.919 (0.818, 1.032)	0.152	0.974 (0.837, 1.135)	0.737	0.768 (0.57, 1.034)	0.082	0.897 (0.739, 1.090)	0.273

**Panel B. Latinx Youth (n=510)**								
	**Ever Initiated Substance Use**		**One Substance**		**Two Substances**		**Three Substances**	
	**HR (95% CI)**	**P Value**	**HR (95% CI)**	**P Value**	**HR (95% CI)**	**P Value**	**HR (95% CI)**	**P Value**
Child Risk Factor	1.435	0.009	1.231	0.273	1.540	0.127	**1.763**	0.045
	(1.096, 1.879)		(0.849, 1.786)		(0.884, 2.681)		(1.013, 3.067)	
Caregiver Risk Factor	1.116	0.368	1.239	0.162	1.182	0.353	0.822	0.345
	(0.879, 1.417)		(0.918, 1.673)		(0.831, 1.680)		(0.548, 1.234)	
Household Risk Factor	0.870	0.516	0.844	0.505	0.769	0.516	1.099	0.825
	(0.572, 1.323)		(0.512, 1.390)		(0.347, 1.702)		(0.475, 2.545)	
Neighborhood Risk Factors								
Neighborhood Social Disorder	1.161	0.044	1.133	0.235	**1.224**	0.041	1.123	0.359
	(1.004, 1.343)		(0.922, 1.393)		(1.008, 1.486)		(0.877, 1.438)	
Neighborhood Violent and Property	0.801	0.082	0.778	0.189	**0.598**	0.025	1.083	0.721
Crime Rates	(0.623, 1.029)		(0.536, 1.132)		(0.383, 0.936)		(0.700, 1.676)	
Neighborhood Social Capital	0.959	0.412	0.993	0.913	0.900	0.262	0.959	0.703
	(0.868, 1.06)		(0.868, 1.135)		(0.748, 1.082)		(0.774, 1.189)	

Unstandardized exponentiated coefficients (HR = Hazard Ratio) are presented in the first column. CI=Confidence Interval. Significant hazard ratios are bolded.

Table S3. Substance Use Initiation during Adolescence by Type of Substance (N = 946).

	Ever Initiated Any		Cigarette		Alcohol		Marijuana		Polysubstance Use	
**Risk Factor**	**HR (95% CI)**	**P Value**	**HR (95% CI)**	**P Value**	**HR (95% CI)**	**P Value**	**HR (95% CI)**	**P Value**	**HR (95% CI)**	**P Value**
Race/Ethnicity (African American = 1)	0.852 (0.609, 1.191)	0.348	0.801 (0.470, 1.365)	0.414	1.029 (0.491, 2.158)	0.940	0.881 (0.437, 1.778)	0.724	0.757 (0.377, 1.517)	0.432
Child Risk Factor	**1.339** (1.105, 1.622)	0.003	**1.396** (1.032, 1.889)	0.030	1.467 (0.961, 2.240)	0.076	1.243 (0.861, 1.796)	0.246	1.175 (0.812, 1.700)	0.392
Caregiver Risk Factor	1.066 (0.896, 1.267)	0.472	1.185 (0.940, 1.493)	0.151	1.057 (0.735, 1.518)	0.766	1.073 (0.732, 1.574)	0.718	0.870 (0.611, 1.239)	0.441
Household Risk Factor	**0.739** (0.559, 0.976)	0.033	0.793 (0.516, 1.219)	0.291	0.662 (0.350, 1.249)	0.203	0.679 (0.385, 1.199)	0.182	0.880 (0.459, 1.688)	0.700
Neighborhood Risk Factors										
Neighborhood Social Disorder	**1.204** (1.092, 1.326)	0.000	**1.184** (1.002, 1.400)	0.047	**1.252** (1.025, 1.529)	0.028	1.175 (0.955, 1.447)	0.128	1.171 (0.930, 1.474)	0.181
Neighborhood Violent and Property Crime Rates	0.872 (0.722, 1.053)	0.155	0.852 (0.614, 1.183)	0.338	0.806 (0.548, 1.186)	0.274	0.875 (0.623, 1.229)	0.442	1.000 (0.679, 1.474)	0.998
Neighborhood Social Capital	0.946 (0.877, 1.020)	0.150	0.882 (0.771, 1.009)	0.068	1.077 (0.920, 1.259)	0.356	0.954 (0.823, 1.106)	0.531	0.929 (0.793, 1.089)	0.366

Unstandardized exponentiated coefficients (HR = Hazard Ratio) are presented in the first column. CI=Confidence Interval. Significant hazard ratios are bolded. Polysubstance use is the initiation of 2 or more of these substances.

Table S4. Substance Use Initiation during Adolescence by Race, Ethnicity and Type of Substance.

**Panel A. African American (n = 436)**										
	**Initiated Any**		**Cigarette**		**Alcohol**		**Marijuana**		**Polysubstance Use**	
**Risk Factor**	**HR (95% CI)**	**P Value**	**HR (95% CI)**	**P Value**	**HR (95% CI)**	**P Value**	**HR (95% CI)**	**P Value**	**HR (95% CI)**	**P Value**
Child Risk Factor	1.269 (0.963, 1.673)	0.091	1.270 (0.830, 1.945)	0.271	1.615 (0.963, 2.710)	0.069	1.019 (0.589, 1.763)	0.948	1.349 (0.830, 2.193)	0.226
Caregiver Risk Factor	0.977 (0.752, 1.270)	0.862	1.139 (0.825, 1.574)	0.430	0.956 (0.508, 1.800)	0.889	1.164 (0.695, 1.951)	0.563	**0.516** (0.290, 0.919)	0.025
Household Risk Factor	**0.626** (0.442, 0.887)	0.008	0.678 (0.406, 1.132)	0.138	0.592 (0.244, 1.434)	0.246	0.604 (0.325, 1.123)	0.111	0.679 (0.239, 1.930)	0.468
Neighborhood Risk Factors										
Neighborhood Social Disorder	**1.229** (1.073, 1.408)	0.003	**1.428** (1.143, 1.784)	0.002	1.043 (0.770, 1.412)	0.786	1.213 (0.926, 1.588)	0.160	1.007 (0.657, 1.544)	0.974
Neighborhood Violent and Property Crime Rates	0.996 (0.746, 1.330)	0.980	0.805 (0.493, 1.315)	0.387	1.206 (0.612, 2.378)	0.588	0.853 (0.510, 1.428)	0.545	1.641 (0.927, 2.906)	0.089

Neighborhood Social Capital	0.919 (0.818, 1.032)	0.152	0.904 (0.722, 1.131)	0.375	0.964 (0.734, 1.268)	0.796	0.986 (0.811, 1.199)	0.887	0.814 (0.652, 1.015)	0.067

Panel B. Latinx Youth (n = 510)

	**Inititated Any**		**Cigarette**		**Alcohol**		**Marijuana**		**Polysubstance Use**	
**Risk Factor**	**HR (95% CI)**	**P Value**	**HR (95% CI)**	**P Value**	**HR (95% CI)**	**P Value**	**HR (95% CI)**	**P Value**	**HR (95% CI)**	**P Value**
Child Risk Factor	**1.435** (1.096, 1.879)	0.009	**1.628** (1.048, 2.529)	0.030	1.305 (0.695, 2.453)	0.408	1.502 (0.951, 2.374)	0.081	1.115 (0.670, 1.855)	0.676
Caregiver Risk Factor	1.116 (0.879, 1.417)	0.368	1.289 (0.937, 1.772)	0.118	0.932 (0.550, 1.579)	0.795	1.018 (0.584, 1.774)	0.950	1.049 (0.684, 1.607)	0.828
Household Risk Factor	0.870 (0.572, 1.323)	0.516	0.960 (0.526, 1.754)	0.895	0.667 (0.219, 2.028)	0.475	0.775 (0.322, 1.869)	0.571	1.059 (0.453, 2.476)	0.895
Neighborhood Risk Factors										
Neighborhood Social Disorder	**1.161** (1.004, 1.343)	0.044	0.961 (0.777, 1.188)	0.712	**1.508** (1.141, 1.994)	0.004	1.127 (0.813, 1.563)	0.474	**1.263** (1.002, 1.591)	0.048
Neighborhood Violent and Property Crime Rates	0.801 (0.623, 1.029)	0.082	0.899 (0.563, 1.437)	0.657	**0.604** (0.387, 0.941)	0.026	0.859 (0.551, 1.340)	0.503	0.823 (0.511, 1.324)	0.421
Neighborhood Social Capital	0.959 (0.868, 1.060)	0.412	0.862 (0.734, 1.012)	0.069	1.194 (0.954, 1.495)	0.121	0.932 (0.764, 1.138)	0.492	0.992 (0.792, 1.243)	0.946

Unstandardized exponentiated coefficients (HR = Hazard Ratio) are presented in the first column. CI=Confidence Interval. Significant hazard ratios are bolded.

Polysubstance is defined as the use of 2 or more of the three substances.
